# Editorial: Plants surviving in extreme environment: harnessing soil-plant–microbial relationship to enhance crop health and productivity

**DOI:** 10.3389/fmicb.2024.1388802

**Published:** 2024-04-25

**Authors:** Priyanka Chandra, Dilfuza Egamberdieva, Nirmalendu Basak, Arvind Kumar Rai, Bandeppa Sonth

**Affiliations:** ^1^Division of Soil and Crop Management, ICAR-Central Soil Salinity Research Institute, Karnal, Haryana, India; ^2^Faculty of Biology, National University of Uzbekistan, Tashkent, Uzbekistan; ^3^Institute of Fundamental and Applied Research, National Research University TIIAME, Tashkent, Uzbekistan; ^4^Division of Soil Science, ICAR-Indian Institute of Rice Research, Hyderabad, Telangana, India

**Keywords:** plant-microbes interaction, abiotic and biotic stresses, plant growth-promoting rhizobacteria (PGPR), soil health, sustainable agriculture

Globally, the sustainability of food production is challenged by biotic and abiotic stressors acting alone or in combination in different agricultural systems. Environmental factors such as high temperatures, droughts, waterlogging, excessive salinity, and other pollutants are the key stressors that limit crop choice and cause significant losses in agricultural productivity. However, crop plants vary in their adaptive response to harsh environmental and edaphic circumstances, but the mutualistic association of the microorganisms with crop plants in diverse environments exhibits enormous metabolic capabilities to mitigate abiotic stresses (Chandra et al., 2022). Microbial partners regulate the systemic and defense mechanisms of crop plants in response to unfavorable external conditions, as they are an essential component of the living ecosystem (Verma et al., 2023). Many rhizobacteria possess the ability to produce 1-aminocyclopropane-1-carboxylate (ACC) and deaminase enzymes to transform ACC into α-ketobutyrate and ammonium, which are responsible for plant growth promotion and stress tolerance. The beneficial rhizobacterial partner mobilizes and/or solubilizes minerals through the production of hydrolyzing enzymes and organic acids. Additionally, they also favor the partner by preventing infection by phytopathogens. The changes in phytohormone, antioxidative enzymes (catalase, superoxide dismutase, and guaiacol peroxidase) and osmolyte (proline and glycine betaine) production by the plant –microbe interaction also provide an adaptive advantage in stressed ecologies.

The aim of this Research Topic was to develop an understanding of plant–microbial relationships under extreme environmental conditions through selected studies. This Research Topic provides a comprehensive and mechanistic view of the microbial response to environmental changes by bringing together 20 studies that illustrate molecular techniques to understand the mechanisms and function of microbial communities through integrated ecological and biogeochemical approaches. In total, 19 original research studies (including field surveys and laboratory or field experiments) and one review article were contributed to this research area. In addition to focusing on niche differentiation in the bacterial microbiomes of the rhizosphere and endosphere, differential metabolite profiles, and the link among soil microbes, this Research Topic also presents new findings on the variables that influence microbial communities, the type and intensity of reactions, and the differences in responses among microbial groups. Yuan et al. offered thorough empirical proof for the significance of silicon fertilizer in agriculture. The phylogenetic and chemotaxonomic studies established the lack of transmissible resistance mechanisms for safe agronomic usage of *Pseudomonas mercuritolerans* under mercury stress conditions (Robas Mora et al.). Singh et al. outlined the taxonomic composition and functional diversity of the potential role of the *Indica* rice rhizome in the development of next-generation microbiome-based technologies for yield enhancement. A review paper by Kumar et al. emphasized the importance of scientific discoveries and policy support for pulse-based cropping systems to achieve the United Nations' Sustainable Development Goals. Two studies reported on the structural features and diversity of rhizosphere bacterial populations of wild *Fritillaria przewalskii* Maxim. (Cui et al.), and *Festuca rubra* subsp. *pruinosa* (Toghueo et al.). Some studies highlighted the importance of soil biological health (Chandra et al.; Chaganti et al.; Rai et al.; Li, Wang et al.; Lu et al.; Yuan et al.) and endophytic bacteria (Badran et al.) for improving productivity, disease resistance and sustainability of different cropping systems and heavy metal contaminated soils (Shahzad et al.).

The response of the microbial flora to different edaphic parameters determines the mitigation capabilities of their mutualistic association with crop plants. The improved performance of wheat and ryegrass in association with halotolerant bacterial and fungal consortia (Marghoob et al.) and *Gracilibacillus dipsosauri* (Li, Zheng et al.), respectively, highlighted the importance of the microbial formulations in reducing salt stress. Adaptation of the pathogenic fungus *Fusarium oxysporum* f. sp. *pisi* by genetic or mutational alterations under the changed temperature, pH, and nitrogen levels are also important factors affecting the disease occurrence and associated management strategies (Chakrapani et al.). The beneficial effect of the bio-stimulants is also modified by the nature of the interaction with other associated microbial communities (Wang et al.; Wei et al.) and the composition of the rhizosphere exudates (Zhao et al.). Although this topic adequately highlights the potential of soil-plant-microbial interaction in extreme environments, further investigations in this domain will enhance the current comprehension of plant-microbial interaction and its effectiveness from agricultural practice perspectives. These studies further validate the importance of plant-microbial interaction, which can play a significant role in boosting agricultural productivity and stress tolerance.

## Author contributions

PC: Writing – original draft, Writing – review & editing. DE: Writing – review & editing. NB: Writing – review & editing. AR: Writing – review & editing. BS: Writing – review & editing.
